# Medical Intelligence

**Published:** 1822-03-01

**Authors:** 


### MEDICAL INTELLIGENCE.

A piece of plate has been presented to Sir Gilbert Blane by several of the senior physicians and surgeons of His Majesty's Navy.

The members of the Medico-Chirurgical Society of London dine together at the Freemason's Tavern, Great Queen Street, on the *fifth* of March, at six o'clock, Dr. Cooke in the Chair. It is expected that there will be a numerous attendance of rank and talent at this anniversary meeting of the Society.

Dr. William Burnett, formerly Physician to the Mediterranean Fleet, and author of the able work on Mediterranean Fever, has been appointed to the Naval Medical Board, as colleague of Dr. Weir. He has been succeeded in his practice at Chichester by Dr. Forbes, of Penzance, the translator of Laennec on Diseases of the Chest.

Dr. Macartney, of Dublin, has for some time employed a solution of alum and nitre for the purpose of preserving anatomical preparations. He finds that it preserves the natural appearances of most parts of the body more completely than spirit or any other fluid hitherto used.—*Medical Repository.*

The Medical Society of London has proposed a gold medal, value 20 guineas, for the best dissertation on Dropsy. The conditions are those usually prescribed on such occasions.

A gentleman in London has lately used the concentrated acetic acid in cases of tinea, as an external application with great success. The Prussic acid has also been found a powerful application in cutaneous affections, but its price is a great objection.

Dr. Ed. Nath. Bancroft, author of the able work on Yellow Fever, who has been dead and buried in the medical journals, is alive and well.

*Suicides.* It appears by a statistical work of M. Fournier, that, in the year 1818, 330 suicidal attempts had been made in Paris, 241 of which terminated in death. During the year 1821, there were but 32 suicides committed in London, whose population is nearly a third more than that of Paris.

*Jacksonian Prize.*—Three of these are proposed by the College of Surgeons this year, viz. Injuries and Diseases of Muscle—Diseases of the Skin—and Diseases of the Rectum. Conditions as usual.

*Mr. Ring.* We have to record this quarter the death of Mr. Ring, well known in the literary and professional world by his different writings. He died of apoplexy.

*M. Laennec.* We are happy to hear that M. Laennec, now so well and so favourably known to the British Profession, is returned to Paris with renovated health, and with unabated zeal for the promotion of pathological science.

*Erysipelas.* Mr. Brodie has experimented on the American mode of treating erysipelas by mercurial frictions, as stated some time ago in this Journal. The utility of the application was unquestionable, but it was attended with the inconvenience of producing salivation sometimes. He therefore substituted simple for mercurial ointment, and found that, in several instances, it appeared to enable the patient to go through the various stages of the disease in a more favourable manner than under ordinary circumstances. *London Med. and Phys. Journal.*

